# Retraction Note: Improving usability and pregnancy rates of a fertility monitor by an additional mobile application: results of a retrospective efficacy study of Daysy and DaysyView app

**DOI:** 10.1186/s12978-019-0728-3

**Published:** 2019-05-14

**Authors:** Martin C. Koch, Johannes Lermann, Niels van de Roemer, Simone K. Renner, Stefanie Burghaus, Janina Hackl, Ralf Dittrich, Sven Kehl, Patricia G. Oppelt, Thomas Hildebrandt, Caroline C. Hack, Uwe G. Pöhls, Stefan P. Renner, Falk C. Thiel

**Affiliations:** Frauenklinik, Universitätsklinikum, Erlangen, Universitaetsstrasse 21–23, 91,054, Erlangen, Germany

The Editor-in-Chief is retracting this article [1]. After publication concerns were raised about the reliability of the estimates of contraceptive effectiveness for the Daysy device when being used together with the DaysyView app [2]. Independent post-publication peer review has confirmed that there are fundamental flaws in the methodology which mean that the conclusions are unreliable due to selection bias and the retrospective self-reporting of whether pregnancies were intentional. The authors do not agree with this retraction. In particular, the authors are of the opinion that the retrospective design of their underlying study is described and that the respective disadvantages are discussed transparently in this article.
